# Ovariotomy; When and How to Operate; after Treatment

**Published:** 1868-01

**Authors:** E. S. Belden


### OVARIOTOMY; WHEN AND HOW TO OPERATE; AFTER TREATMENT.

Abstract from Remarks of E. R. PEASLEE, M.D., at N. Y. Medical Journal Association, May 24, 1867.

REPORTED BY E. S. BELDEN, M.D.

MR. PRESIDENT—Three years ago, I presented a paper to the Academy of Medicine on Ovariotomy, which was published in its "Transactions." My opinions as there expressed have not materially changed; but as some new things have been proposed since then, it is to these especially that I wish to call your attention this evening.

At that time there still remained some doubt in the minds of certain eminent surgeons whether ovariotomy should be recognized as a legitimate surgical operation or not. I then gave my reasons, and the statistical facts and arguments for the conclusion that it has as high a claim to be thus recognized as any other important operation, and I think very few would now be found to controvert it. During the past five years the operation ovariotomy in the hands of experienced ovariotomists has done more in proportion to the number of cases operated upon, for the prolongation of life, and less to shorten life than any other surgical operation which can be at all compared with it in point of magnitude. The percentage of lives saved by experienced ovariotomists during this time is probably not short of eighty per cent.; and these are patients who are doomed on an average to die without the operation within twelve to eighteen months, and, who, if they are saved thus, are likely to live as long as other women of the same age.

A point then in connection with ovariotomy, of much interest is, that the operation is followed in a very few days, either by death, or, on the other hand, by life and health of indefinite duration, as just stated. If successful, it is to the woman a resurrection. A patient remarked to me, "she felt as though she had commenced an entirely new life." The results suggest the expression of the poet:—

"Aut cita mers, aut victoria læta."

If this operation is performed in a case which is uncomplicated, it is one of the most simple of surgical operations. On the other hand, if complicated in a high degree, it is the most difficult and formidable operation the surgeon ever attempts to perform. And not even the most experienced operator can certainly determine beforehand, whether he has a simple or a complicated case. Where he had expected no difficulty at all, he may find a condition of things that will require all his coolness, deliberation, and caution, to enable him to get through without leaving his patient dead upon the table. I can say that ovariotomy sometimes demands more of all these qualities, and more care and judgment in the subsequent treatment, than any other I have ever attempted: though, during seventeen years of my professional life, I frequently performed all the capital operations. This peculiarity has not, however, been sufficiently recognized.

Encouraged by the facility of operation, and the frequent successes of the most favorable cases, many a physician has attempted ovariotomy, who would never think of attempting any other surgical procedure, not even the amputation of a finger. The results have been, as might be anticipated; the first case of real difficulty, or perhaps the second, terminating alike the operator's zeal and his success. It is, however, the fact that a simple case operated on at the most favorable time, will probably recover in spite of a large amount of operative bungling.

We all know the difficulty of diagnosis, in complicated cases, and the mistakes which have been made, but I omit this topic entirely.

The first question I will consider is, at what period in the development of the disease shall the operation be performed? The question lies, of course, between performing the operation early, while the patient is still in robust health, and deferring it until she begins to be somewhat reduced by the disease; no one would defer til she is just about to die in consequence of it.

Spencer Wells, and Baker Brown, maintain that the operation should be performed early, in full health. Mr. Hutchinson, of London, says it should be performed "as early as possible." Baker Brown once performed this operation when the tumor had been detected only eight weeks before. The patient died on the ninth day. Mr. Erichsen, Tyler Smith, and Dr. W. L. Atlee, of Philadelphia, take the ground that this operation should not be performed at this time; but when at length the patient begins to yield to the disease; and in this opinion I concur.

The reason in general, for the first proposition, is that the patient endures a severe operation better if it is performed when she is in full health; a statement which I have not found to be substantiated by statistics—as shown in the paper to which I have alluded.

I cannot here specify all my reasons for deferring the operation till the general health begins to fail, but the following are some of them:

In the first place, if the operation is performed upon a patient in full health, she is, other things being equal, more liable to peritonitis after it; and peritonitis destroys about one-fourth of all who die from the effects of the operation. Spencer Wells, operating on patients in full health, when symptoms of peritonitis appear, bleeds them, and in that way has sometimes saved them. And certainly this is very judicious practice, if the operation is performed thus early; but I think it would be better to diminish the risk of peritonitis, by some delay. Besides, if the patient is in good health, she is certainly in no immediate danger; and we make sure of adding a certain amount of time to the patient's life by the delay. And we may often wait six months or a year, and find her still in as good health as to-day.

Again, if we wait, further opportunity is given to perfect the diagnosis; and every one knows how difficult this is in some cases. Even Spencer Wells, whom I saw perform his 174th and 175th operations last July, and who has now operated over 200 times, still pronounces his diagnosis with caution. But he waits and reëxamines the case until he feels very positive; and he has very seldom had to record a mistake. If we wait till the abdomen is largely distended with fluid, it may become necessary to tap her, though still in pretty good health; and this operation may at once clear up all doubt, if any before existed, whether the case be one of ovarian tumor. If the tumor be one which can be very much diminished by tapping,

*i.e.*, if there be one or more large sacks—I make it a rule to tap before deciding as to the operation of ovariotomy. Many of the tumors in this region, at first thought to be ovarian, are not so, but are cured by a single tapping. I have had two such cases, and consider this a very important point.

A patient, the wife of a professor in one of the Western colleges, called on me some years since, who had seen three or four of the most distinguished surgeons and physicians in this city, all of whom had pronounced her case one of ovarian tumor. I examined her case thoroughly, and had not the least doubt that it was such. She went to a distinguished surgeon in Massachusetts, who had no doubt as to its nature, and offered to remove it immediately if she wished. I declined to do anything in the case till tapping should be required; after which it would be time to decide respecting ovariotomy. A year afterward, the tumor began to interfere with respiration and digestion, and I considered that the time for tapping had come. I tapped her, but there was no ovarian tumor; and no farther operation was required. The uterus, prolapsed by the pressure of the fluid, soon regained its normal position, under appropriate treatment. She gave birth to a child about seventeen months afterwards; and has now enjoyed perfect health for the last five years. The sack was one of those developed in the broad ligament.

I have had another case like the preceding; and by waiting and tapping, I found a similar sac, instead of an ovarian tumor, and that no further operation was required.

Spencer Wells has noted a few cases where he had tapped tumors of this kind with the same result.

The fluid contained in these sacs is as transparent as water, and contains no albumen, (certainly in most cases,) and has great refractive power. It is entirely different from the ovarian fluid; especially in the fact of the absence of albumen.

Another reason for waiting, is, that the success of the operation is greater, other things being equal, if the tumor is *large;* and this for two reasons, I would not be willing to remove an ovarian tumor that was of the size of a fœtal head in a person of ordinary health.

1. If the tumor is large, by its constant pressure upon the peritoneum, the latter is rendered more insensible to irritation and consequent inflammation.

2. And another reason for not operating when the tumor is small, is, that if after the incision is closed up, if the patient should vomit or cough, there may be trouble from straining the muscles, or even from a hernial protrusion through the wound. The latter occurred in one instance where I removed a fibrous tumor of the uterus, and the patient died in consequence. She had a severe cough at the time of the operation, and which she had concealed from me by taking opium, knowing that I would not operate if I discovered it. Three or four hours after the operation she began to cough violently. This I could not control; and the consequence was, a hernial protrusion between the needles, although they were but half an inch apart. As I was sixty miles from the patient when the hernia occurred, the bowel mortified before it was reduced, and she died six days after the operation.

As an argument against the assertion that a person in full health bears this operation, or any severe operation, better than when somewhat reduced in health, consider the following fact. If we divide the amputations of the lower extremity into two classes; first, operations performed upon persons in full health, as for elephantiasis, or in consequence of accidents, etc., called amputations of expediency or of necessity; and second, operations, performed upon patients somewhat exhausted by disease, called expediency, pathological amputations, as those for disease of the joints, etc. It has been found that while in patients of the first class (amputations upon persons in full health), 42 per cent. die; in amputations of the second class, only 14 per cent. are fatal. An operation upon an ovarian tumor, while the patient is in full health, is literally as well as logically an operation of expediency; and here, as with amputations, the best time for operating is, when the health of the patient is somewhat reduced. No ovariotomist has had better success than Dr. Tyler Smith, and he acts upon this principle, as I have before stated.

I have now under my observation some ten cases; some of which I have kept waiting a year or more; while others who, determined to have the operation performed, have found those willing to operate, have succumed. I have not yet had to regret deferring the operation, as I have explained.

In regard to the operation of ovariotomy itself, I can here only consider the incision; and the manner of treatment the pedicle of the tumor removed. In regard to the incision, the rule is, that it at first should not be more than one or two inches long, through the peritoneum, but somewhat longer, of course, through the skin, etc., than internally. Next I pass a steel bougie into the peritoneal cavity, and around the tumor, if possible, to ascertain if there are any adhesions. Afterward the incision is to be enlarged, or not, as may be required; the rule being to leave it as short as will answer the purpose. It should, however, always be regarded as merely explorative, until the operator has decided that the tumor is to be removed. If he finds that there are extensive adhesions, or especially if to the alimentary canal, uterus, or bladder, it is very much better to make a long incision at once, that the adhesions can be seen before they are torn across; else they may be torn from the intestines, bladder, or uterus, instead of from the tumor, and troublesome hemorrhage may take place into the cavity of the peritoneum.

The tumor having been taken away, how shall we treat the pedicle? If the pedicle were in no danger of bleeding after its division, all operators would agree that it should be returned into its natural position, and the wound be closed up. But the *only* thing that we can rely upon, to prevent hemorrhage from the pedicle to the greatest possible certainty, is a ligature tied in a knot. If we have tied it tight enough to stop the circulation there, even for a few minutes, we may feel very sure it will serve us, unless it subsequently slips off. I shall consider other methods further on.

But if the legature is used, then the question arises whether it should be cut off short or left hanging out of the wound. The objection of Spencer Wells, to the ligature, is, that it always produces a sloughing of the stump of the pedicle. (The ligature being doubled and carried through the middle of it, one half is inclosed on each side.) "If it is cut off short, the result will inevitably be," he thinks, "that the ligature and the stump of the pedicle will slough off and remain in the abdominal cavity." But if the ligature is not cut short, of course it will be all the same so far as the slough is concerned.

Spencer Wells has even suggested the idea that, on the whole, it would be better to leave these ligatures coming out of the lower end of the wound; since inasmuch as there must be a slough, the dead, putrefying matter thus formed in the abdominal cavity, in this way, finds a conduit, which, by capillary attraction will drain it off.

Dr. Bouth, who also takes the same view, made some experiments upon the lower animals, and found that dead meat, even if fresh, in the abdominal cavity produced a low putrid fever, of which they died.

It is, however, a fact, that as large a proportion of the women treated in this way, by ligature, have recovered, as of those treated by other methods; and it is also true that most of them had no symptoms of low fever. None of my patients have had any such symptoms, except as evidently produced by other causes, and I have always used the ligature.

Dr. Tyler Smith operated upon eight patients, with the ligature cut short, and they recovered without any symptoms of low fever at all.

And the very fact that patients do recover thus without fever, shows that no absorption of putrid matter has taken place, and therefore that no such has been formed. This was the conclusion I arrived at three years ago, when I read the paper to which I have referred. I have since had opportunity to demonstrate its correctness.

My first six cases were treated with ligatures, the end hanging out of the incision, and they all recovered. Since then, I have had an opportunity of examining two of my own cases that terminated fatally, in which I applied the ligature, and cut it short. They both died seventeen days after the operation. I have also examined another case lost, by another operator, after similar treatment of the pedicle. In none of these cases did any slough occur. In one instance, the ligature had actually cut off the portion which was included in it. In another, it had cut it to that extent, that there was left only enough to half fill the loop; and in a third, the ligature was so entirely covered up, that I could with difficulty find it. But in every case there had been an exudation of plasma over the stump and ligatures, which had nourished the part which was beyond the ligature, and attached it to the living tissue in its neighborhood. I state, therefore, without any hesitation, that I consider the point demonstrated, that there is no slough of the pedicle when we put a ligature around it, as I have explained. And, if there is no slough, what is the use of leaving one end of it hanging out of the wound? It seems to me, at the present time, therefore, that the best way to treat the pedicle is, to apply the ligature, cut it short, and close up the whole incision. Still, Spencer Wells most frequently used the clamp, though he recently stated that he is not yet decided which is the best way to treat the pedicle. I cannot here speak of the relative merits of the clamp, nor can I recommend the ecraseur, though it has several times succeeded.

The actual cautery has been applied to the pedicle by several operators, but more frequently, of late, by Baker Brown. Nearly a year since, I saw him perform his 101st and 102d operation of ovariotomy, and 32d and 33d, in which he had applied the actual cautery to the pedicle. In one of the two cases, the bleeding was not arrested by the hot iron; and he then applied the ligature in the common way, and cut it short. The same has been done in several previous cases; and, of course, all these should have been reported as cases of treatment with the ligature, and not with the actual cautery.

Finally, it may, in time, be demonstrated that the ligature is preferable in one class of cases, the clamp in another, and the actual cautery in a third. Meantime, I hold this up to the present time, the ligature, as an exclusive method, is to be preferred.

Before closing up the wound, it is a question of much importance whether the blood, and other fluid emptied into the peritoneal cavity shall be sponged out or not. My opinion is decidedly that it should be. In every case of my own, I have undertaken to remove every drop of fluid. I lost one patient from septicæmia, produced by a small amount of blood becoming decomposed, which flowed from the omentum after the operation. I see no reason to doubt that the patient would have recovered if the blood had not been there. I have had three cases in which the patients must have died, if I had not reöpened the incision, and washed out the putrified fluid in the peritoneal cavity. My idea has long been, and still is, that where, on the one hand, there is no danger in putting into the peritoneal cavity, a sponge, first dipped in warm water; on the other hand, it may be of the greatest importance to do this. The peritoneum should be included in the sutures closing up the incision. If we leave the edges of the peritoneum gaping, as they will do otherwise, we will have a granulating surface, and the intestines, wherever they touch this surface, become adherent to it. Experiments on the lower animals have shown this. Then, when the pus forms, it falls into the peritoneal cavity and septicæmia will probably ensue. But I close with a few remarks on the after treatment.

I consider that there is much more responsibility attached to the after treatment of a case of ovariotomy, unless it is a very complicated one, than there is to the operation itself. I suppose I have been urged to perform this operation at least one hundred times, where I have declined, because the circumstances forbid my taking charge of the after treatment. In assuming the after treatment of a case, I consider that I am incurring at least three-fourths of the responsibility, and nine-tenths of the anxiety.

In regard to opiates, I think just enough should be given to keep the patient free from pain, and comfortable; not enough to stupefy. I like the action of McMunn's Elixir of Opium, better than any of the other opiates, administered by the rectum if the stomach is irritable. I do not use strong doses of opium as soon as the operation is over, as I consider this objectionable. The patient should be allowed to regain her consciousness as soon as the operation is over. The operation, as well as the ether, has been depressing enough, and nothing more of the kind should be given until we see whether the patient is going to rally or not, and then is the time to give the opium. I have sometimes given a patient only thirty drops of McMunn's Elixir for the first two or three nights, and no more during the treatment. Baker Brown and Spencer Wells have given up the idea of large doses of opium immediately after the operation.

After the first week is over, very little is to be done, under ordinary circumstances. If septicæmia sets in from decomposed fluid in the peritoneal cavity, I would reöpen the incision. I know of no better treatment sufficient to allow the introduction of an elastic bougie, and inject blood-warm water, and let it at once return. The mixture of water and the putrid fluid cannot be as injurious as the fluid alone. The first time that I acted on this idea (in 1855) the patient was stupified by the poison. I injected one quart of water, and she immediately looked up and said she felt as though she had taken a bath. I relieved her in this way every day for a week, using two quarts to a gallon of water at a time. When I found the fluid very fetid, I used the *liquor sodæ chlorinatæ*, in the proportion of two drachms to the pint of water; finally, it returned without any odor at all, and from that time commenced a perfect recovery. In another case I resorted to these injections once or twice daily for fifty days; and, in a third case, I injected one hundred and thirty-five times in seventy-eight days. These three patients recovered, though I feel positive they would all have died, had not the decomposing matter been washed out of the peritoneal cavity. I also combined with the injections, the sulphate of quinine, and the hypo-sulphite of soda.—*Medical and Surgical Reporter.—South. Jour. Med. Sciences.*

